# An Evolutionary Perspective on Linoleic Acid Synthesis in Animals

**DOI:** 10.1007/s11692-017-9436-5

**Published:** 2017-10-23

**Authors:** Miriama Malcicka, Bertanne Visser, Jacintha Ellers

**Affiliations:** 1 Department of Ecological Sciences, Section Animal Ecology, Vrije Universiteit, Amsterdam, De Boelelaan 1085, 1081 HV Amsterdam, The Netherlands; 2 Evolutionary Ecology and Genetics Group, Biodiversity Research Centre, Earth and Life Institute, Université Catholique de Louvain, Croix du Sud 4-5, 1348 Louvain-la-Neuve, Belgium

**Keywords:** De novo synthesis, Arthropods, Essential nutrients, Fatty acids, PUFAs, Desaturase

## Abstract

The diet of organisms generally provides a sufficient supply of energy and building materials for healthy growth and development, but should also contain essential nutrients. Species differ in their exogenous requirements, but it is not clear why some species are able to synthesize essential nutrients, while others are not. The unsaturated fatty acid, linoleic acid (LA; 18:2n-6) plays an important role in functions such as cell physiology, immunity, and reproduction, and is an essential nutrient in diverse organisms. LA is readily synthesized in bacteria, protozoa and plants, but it was long thought that all animals lacked the ability to synthesize LA de novo and thus required a dietary source of this fatty acid. Over the years, however, an increasing number of studies have shown active LA synthesis in animals, including insects, nematodes and pulmonates. Despite continued interest in LA metabolism, it has remained unclear why some organisms can synthesize LA while others cannot. Here, we review the mechanisms by which LA is synthesized and which biological functions LA supports in different organisms to answer the question why LA synthesis was lost and repeatedly gained during the evolution of distinct invertebrate groups. We propose several hypotheses and compile data from the available literature to identify which factors promote LA synthesis within a phylogenetic framework. We have not found a clear link between our proposed hypotheses and LA synthesis; therefore we suggest that LA synthesis may be facilitated through bifunctionality of desaturase enzymes or evolved through a combination of different selective pressures.

**Electronic supplementary material:**

The online version of this article (doi:10.1007/s11692-017-9436-5) contains supplementary material, which is available to authorized users.

## Introduction

Among all the nutritional compounds that make up an organism’s body, many are required as a nutritional supplement, i.e. organisms cannot synthesize these metabolites themselves. Lack of an external resource for such essential molecules can cause a range of deficiency diseases, for example in the case of vitamin, amino acid or mineral deficiencies (Anderson and Connor 1989; Brock and Chapple 2016). The exogenous requirement for essential nutrients is, however, not universal among organisms. Some taxonomic groups, such as microorganisms and plants, are to a large extent autotrophic and can synthesize vitamins, amino acids, and lipids de novo from simpler compounds. Metazoan species, in contrast, are generally heterotrophic for many metabolites, as they lack key enzymes of essential metabolic pathways (Ellers et al. 2012). Metazoans thus generally need regular dietary intake of essential micronutrients to prevent deficiency diseases. It remains to be resolved, however, why some species are able to synthesize essential nutrients, while others are not. One possible explanation is that for some species essential nutrients are abundantly available in their environment. High consumption of essential nutrients may relax selective pressures on an organism to maintain autotrophy for such metabolites (Pandey et al. 2015). Furthermore, if nutrients are supplied by a symbiotic partner or are present abundantly in the environment, de novo synthesis of the nutrient in question becomes redundant and prone to loss or degradation (Visser and Ellers 2008; Ellers et al. 2012; Helliwell et al. 2013). The presence of excess amounts of vitamin C in the diet of primates and other mammals, for example, is thought to have rendered de novo synthesis obsolete and mutation accumulation was found to compromise functioning of the gene underlying vitamin C production (Chatterjee 1973; Ohta and Nishikimi 1999). Similarly, some genes required for the biosynthesis of vitamin B_6_ were lost after the divergence of vertebrates and invertebrates, leaving all mammals unable to synthesize vitamin B_6_ (Kennedy 2016).

One class of essential nutrients is found among polyunsaturated fatty acids (PUFAs). PUFAs are long chain fatty acids with multiple double bonds that are vital for body functions, such as the formation and functioning of cell membranes, as well as the immune system. PUFAs further play an important role in cell physiology, signaling and reproduction (Belury 2002; El-Yassimi et al. 2008; De Veth et al. 2009), and in humans PUFAs lower the risk of coronary artery disease, some nerve diseases like Alzheimer’s, schizophrenia and metabolic syndrome (Booth-Kewley and Friedman 1987; Kalmijn et al. 1997; Horrobin 1998; Hulbert et al. 2005). Some PUFAs have been recognized in animals as essential nutrients for which a dietary source is required (Sinclair et al. 2007; Grosso et al. 2016). The critical step in PUFA biosynthesis is the introduction of a second double bond in a mono-unsaturated fatty acid leading to the formation of linoleic acid (LA). This enzymatic step is carried out by specific desaturases, which were previously thought to be restricted to bacteria, protozoa and plants (de Renobales et al. 1986). Louloudes et al. (1961) were one of the first to show that an insect, the American cockroach *Periplaneta americana*, was able to biosynthesize LA de novo. Since that time, a growing body of research on LA and other PUFAs has confirmed that LA can be synthesized by a range of different insects (de Renobales et al. 1986; Cripps et al. 1986; Aboshi et al. 2013; Shimizu et al. 2014) and other invertebrates, including nematodes and pulmonates (Weinert et al. 1993; Wallis et al. 2002). Consequently, these findings have challenged the long-held assumption that animals lack the ability to synthesize LA de novo.

We currently have a poor understanding of the evolutionary dynamics of the ability for LA synthesis. Until now LA synthesis is not commonly found, but species that are able to produce LA are found in a diverse set of taxonomic groups. A phylogenetic analysis of the pattern of presence and absence of biosynthetic ability of LA is therefore needed to distinguish between different potential evolutionary scenarios. One hypothesis is that a conserved loss of LA synthesis early in the evolution of the Animalia was followed by multiple independent reversals of the trait. Alternatively, the ancestral trait of LA synthesis may have been retained across most families, but many independent losses of the trait were incurred in more shallow evolutionary branches. Clearly, either of these scenarios will involve multiple evolutionary transitions, which suggests that LA biosynthesis is an evolutionarily labile trait. The question is which genetic pathways enable this high evolvability and what convergent selection pressures are leading to this repeated pattern of (re)gain and/or loss. In this review we develop a new perspective on the evolutionary dynamics of LA synthesis. We will (1) compile known cases of LA synthetic ability across the animal kingdom and use a phylogenetic approach to explore macro-evolutionary patterns; (2) review the biochemical and genetic pathways involved in LA synthesis to compare LA synthesis mechanisms in animals to that in plants and microbes; and (3) present new prospects on the biological function of LA synthesis, including potential selective forces leading to gains or losses of the ability to produce LA.

### LA Synthesis Ability and Evolutionary Transitions in Animals

**Fig. 1:**
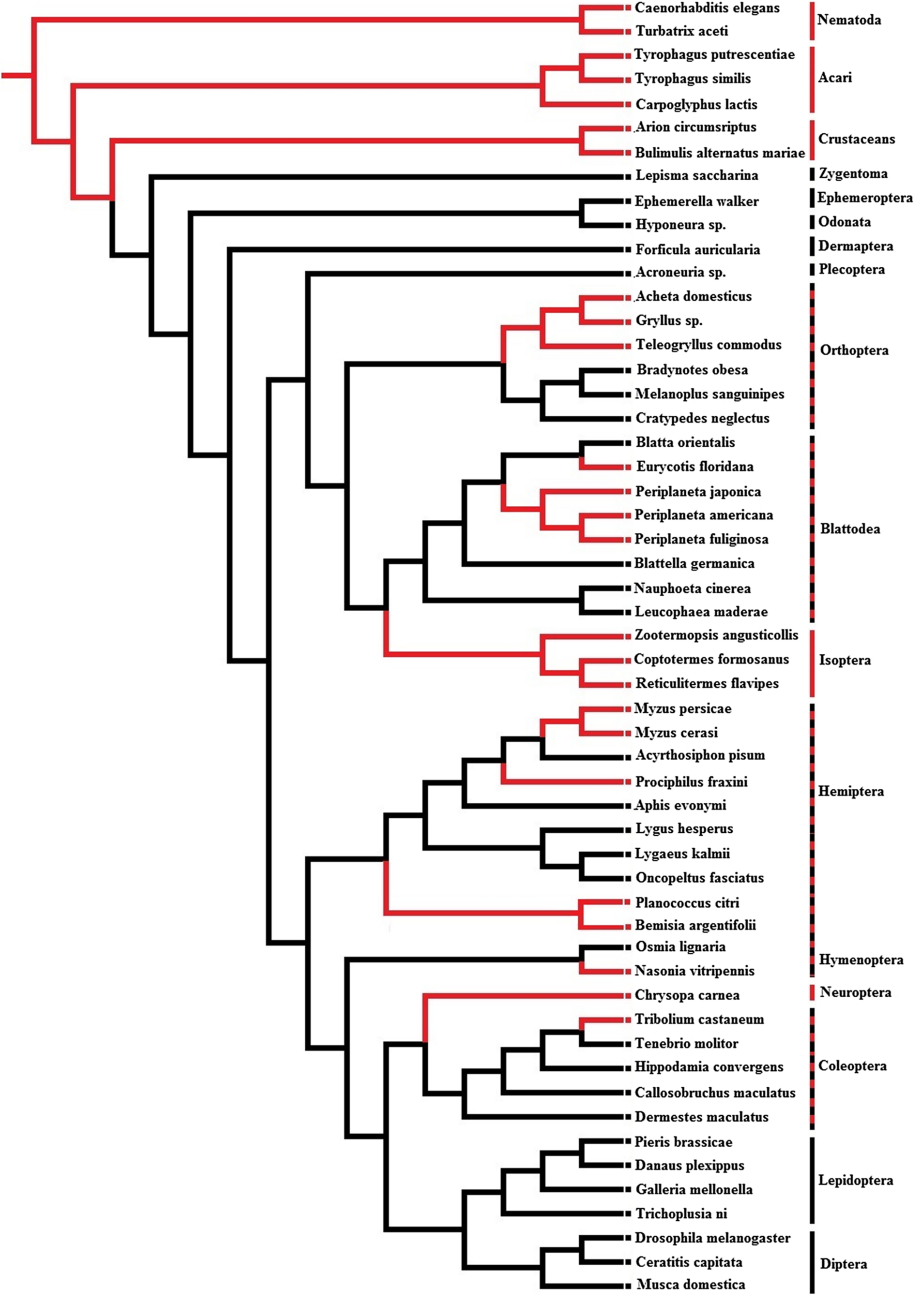
A phylogenetic tree reconstructed based on molecular and morphological data (see Supplementary material 2) of species able (red branches) and unable (black branches) to synthesize LA de novo. (Color figure online)

To gain more insight into the evolution of LA synthesis, we have compiled all available information on the ability to produce LA for invertebrates and reconstructed their phylogeny (Fig. 1; Supplementary information 1). Until now, 54 invertebrate species have been tested for their ability to synthesize LA and over 40% of these species were found capable of producing LA de novo (Fig. 1; Table 1). Within the primitive invertebrates Crustacea, Nematoda and Acari, all seven species tested were found to synthesize LA, revealing that the more ancestral invertebrate groups have functional LA synthetic pathways. The ability for LA synthesis was absent in more primitive insect groups, including Zygentoma, Ephemeroptera, Odonata, Dermaptera, and Plecoptera, although only few species within these groups were tested (5 species in total). Of all insects tested, almost 60% of species were found incapable of producing LA de novo. The other 40% did produce LA, but LA synthesis ability seems to be scattered across the different insect orders.

**Table 1:** Overview of species tested for the ability to synthesize linoleic acid (LA) with references (see Supplementary material 2)

Order	Species/references	LA ability	Sex	Diets	Distribution
Rhabditida	*Turbatrix aceti* ^1,2,3^	+	S	Microbes	?
	*Caenorhabditis elegans* ^4^	+	S, H	Microbes	Temperate
Sarcoptiformes	*Tyrophagus similis* ^5^	+	S	Omnivorous	Temperate
	*Tyrophagus putrescentiae* ^5^	+	S	Omnivorous	Temperate
	*Carpoglyphus lactis* ^6^	+	S	Omnivorous	Temperate
Crustaceans	*Arion circumscriptus* ^7^	+	H	Herbivore	Temperate
	*Bulimulus alternatus* ^7^ *mariae*	+	H	Herbivore	Tropics
Zygentoma	*Lepisma saccharina* ^8^	−	S	Omnivorous	Temperate
Ephemeroptera	*Ephemerella walker* ^8^	−	S	Herbivore	Temperate
Odonata	*Hyponeura* sp.^8^	−	S	Carnivorous	Temperate
Dermaptera	*Forficula auricularia* ^8^	−	S	Herbivore	Temperate
Plecoptera	*Acroneuria* sp.^8^	−	S	Carnivorous	Temperate
Orthoptera	*Acheta domesticus* ^9,10,11^	+	S	Omnivorous	Temperate
	*Gryllus sp*.^8,12^	+	S	Omnivorous	Temperate
	*Teleogryllus commodus* ^13,14^	+	S	Herbivore	Temperate
	*Bradynotes obesa* ^8^	−	S	Herbivore	Temperate
	*Melanoplus sanguinipes* ^8^	−	S	Herbivore	Temperate
	*Cratypedes neglectus* ^8^	−	S	Herbivore	Temperate
	*Blatta orientalis* ^8^	−	S	Omnivorous	Cosmopolitan
	*Eurycotis floridana* ^15^	+	S	Omnivorous	Temperate
	*Periplaneta americana* ^9,10,11,16,17,18^	+	S	Omnivorous	Cosmopolitan
	*Periplaneta fuliginosa* ^8^	+	S	Omnivorous	Temperate
	*Periplaneta japonica* ^8^	+	S	Omnivorous	Temperate
	*Blattella germanica* ^10,19^	−	S	Omnivorous	Cosmopolitan
	*Nauphoeta cinerea* ^8^	−	S	Omnivorous	Tropics
	*Leucophaea maderae* ^8^	−	S	Omnivorous	Tropics
Isoptera	*Zootermopsis angusticollis* ^9,10^	+	S	Lignivore	Temperate
	*Coptotermes formosanus* ^9^	+	S	Omnivorous	Temperate
	*Reticulitermes flavipes* ^9^	+	S	Lignivore	Temperate
Hemiptera	*Myzus cerasi* ^8^	+	S	Herbivore	Temperate
	*Myzus persicae* ^20^	+	S	Herbivore	Cosmopolitan
	*Prociphilus fraxini folli* ^8^	+	S	Herbivore	Temperate
	*Acyrthosiphon pisum* ^10,19^	−	S	Herbivore	Temperate
	*Aphis evonymi* ^21^	−	S	Herbivore	Temperate
	*Planococcus citri* ^8^	+	S	Herbivore	Cosmopolitan
	*Lygus hesperus* ^8^	−	S	Herbivore	Temperate
	*Lygaeus kalmii* ^8^	−	S	Herbivore	Temperate
	*Bemisia argentifolii* ^22^	+	S	Herbivore	Tropics
	*Oncopeltus fasciatus* ^8^	−	S	Herbivore	Temperate
Hymenoptera	*Osmia lignaria* ^8^	−	S	Pollen	Temperate
	*Nasonia vitripennis* ^23^	+	S	Omnivorous	Temperate
Neuroptera	*Chrysoperla carnea* ^8^	+	S	Carnivorous	Cosmopolitan
Coleoptera	*Tribolium castaneum* ^24^	+	S	Herbivore	Cosmopolitan
	*Tenebrio molitor* ^8^	−	S	Herbivore	Temperate

LA ability: + (capable of LA biosynthesis); − (not capable of LA biosynthesis). Mode of reproduction: S (sexual); H (hermaphroditic). Diet: Microbes; Pollen; Herbivore (feeding on plants); Omnivore (feeding on a variety of food of both plant and animal origin); Carnivore (feeding on other animals); Lignivore (feeding on wood-decay material); Frugivore (feeding on fruit). Distribution: temperate climate (between the Tropic of Cancer and the Arctic Circle in the northern hemisphere, and the Tropic of Capricorn and the Antarctic Circle in the southern hemisphere; having 4 seasons); Tropics (according to the Köppen climate classification, a non-arid climate in which all 12 months have mean temperatures of at least 18 °C); Cosmopolitan (across all or most of the world)

Character tracing of LA synthesis in insects suggested that the ability for de novo synthesis in 18 species is a secondarily derived character rather than an ancestral trait (Fig. 1), particularly because LA synthesis is absent from the primitive invertebrate groups. If we assume absence of LA synthetic ability to be the ancestral state in the insects, as a result of convergent evolution (homoplasy) LA synthesis could have evolved eight times independently in different lineages, and lost in two insect groups (Lepidoptera, Diptera). Overall, with the exception of flies and butterflies, the ability for LA synthesis re-evolved in all major insect orders. In Blattodea and Hemiptera the evolutionary history of LA synthesis is equivocal. For the cockroaches, LA synthesis may have been regained once in the common ancestor of *Periplaneta* sp. and *Eurycotis floridana* and subsequently lost again in *Blatta orientalis*. Alternatively, LA synthesis was regained on two separate occasions, once in *E. floridana* and once in the common ancestor of *Periplaneta* sp. The same holds true for the hemipteran clade where LA synthesis could have been regained on two separate occasions or was regained once and subsequently lost again in some species. There is clear support for the hypothesis that a conserved loss of LA synthesis early in the evolution of the Animalia was followed by multiple independent reversals of the trait. To better comprehend those evolutionary transitions, we should look into the biochemical and genetic pathways underlying LA synthesis in these species.

### Biochemical and Genetic Pathways Underlying LA Synthesis

**Fig. 2:**
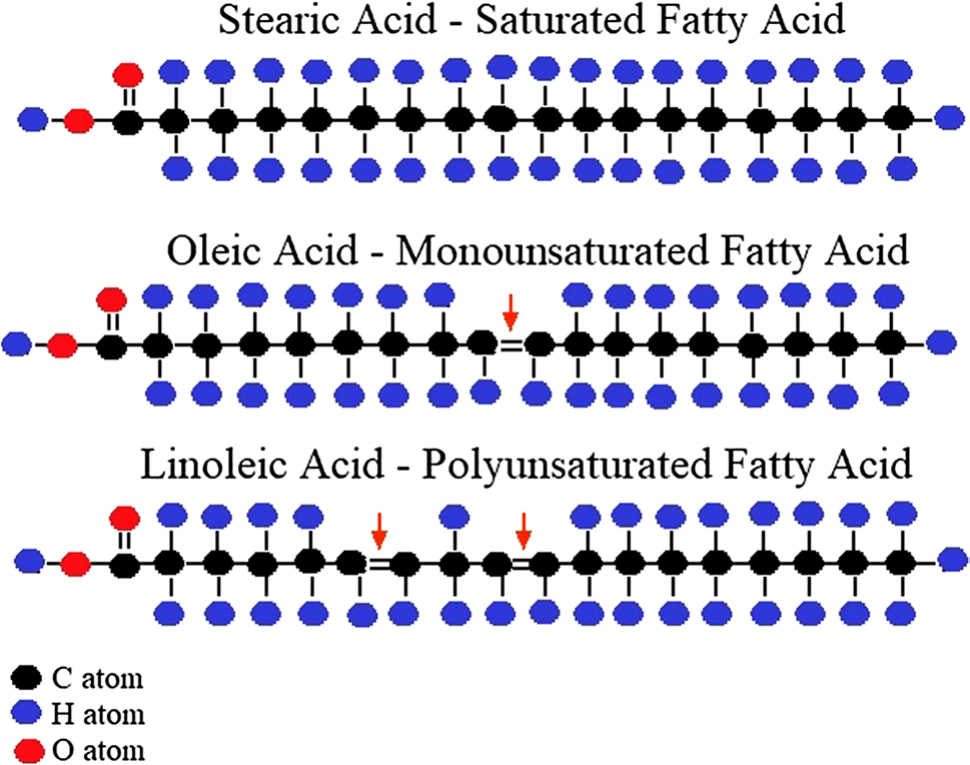
A schematic overview of the fatty acid desaturation mechanism

**Fig. 3:**
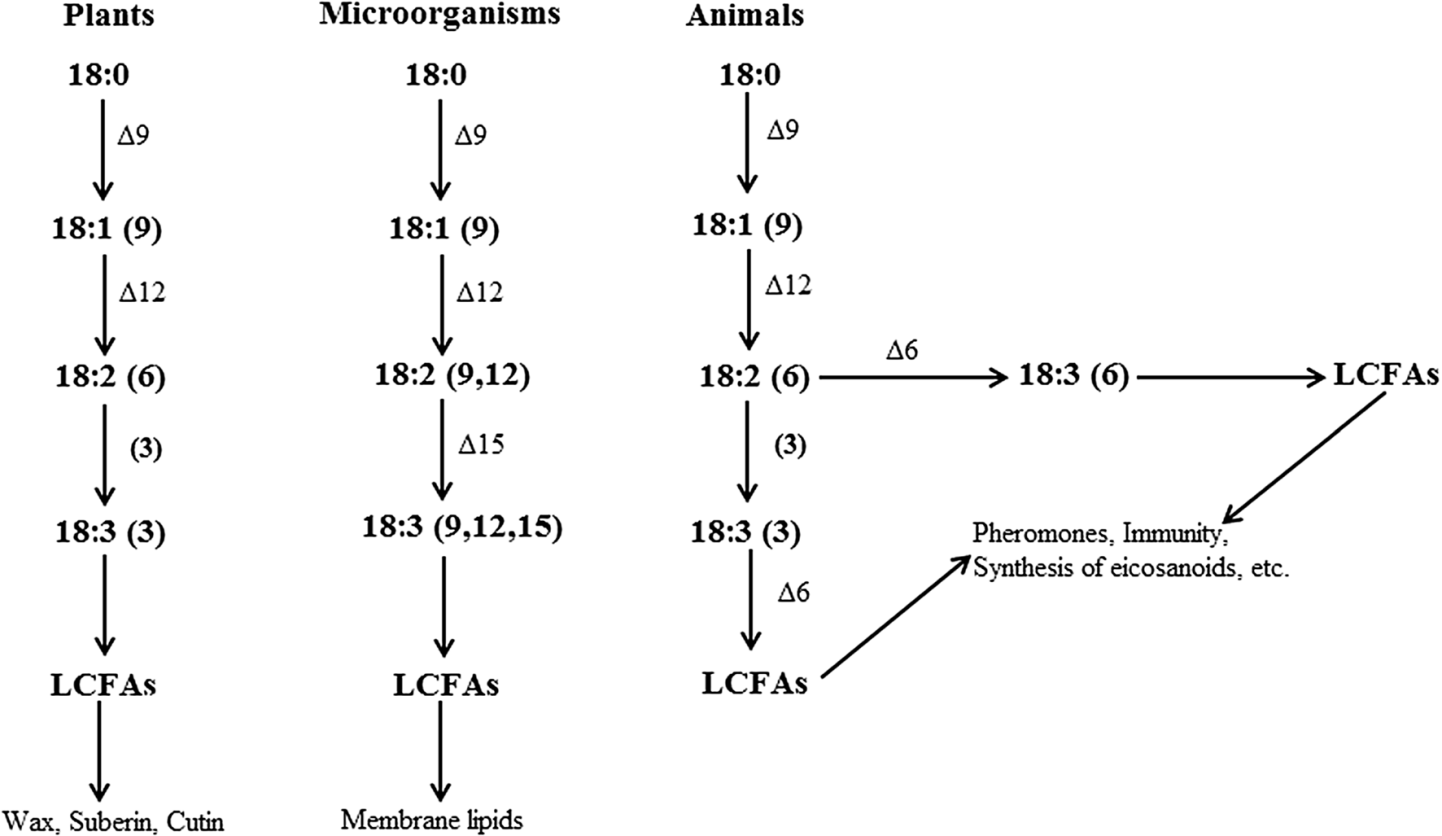
Three types of desaturases are involved in introducing double bonds to long chain FAs with their final products in plants, microorganisms and animals: acyl-ACP, acyl-lipid and acyl-CoA desaturases, respectively (Murata and Wada 1995). Each double bond is indicated by Δx or by the number in brackets, where the double bond is located on the xth carbon–carbon bond, counting from the carboxylic acid end. For example in microorganisms, oleic acid, C18:1 (9) already contains a bond at the 9th carbon position and a bond at the 12th carbon position is added to form linoleic acid, C18:2 (9, 12)

Fatty acid desaturases are enzymes that introduce a double bond between carbon atoms and remove a hydrogen atom, thereby creating unsaturated fatty acids (FAs) (Fig. 2). Three types of desaturases are involved in placing double bonds in long chain FAs: acyl-lipid, acyl-ACP and acyl-CoA desaturases (Fig. 3; Murata and Wada 1995). Acyl-lipid desaturases are found in cyanobacteria and some plants, where bonds are introduced into fatty acids that are in a lipid-bound form (Murata and Wada 1995; Los and Murata 1998). Acyl-ACP desaturases are present in plant plastids and introduce double bonds to lipids bound to an acyl carrier protein (ACP) (Murata and Wada 1995). Acyl-CoA desaturases are found in fungal and animal cells in which double bonds are introduced involving coenzyme A (CoA) (Macartney et al. 1994). PUFAs can be synthesized through the action of a range of desaturases, generating different products depending on the location at which double bonds are introduced in a long chain FA molecule (Wallis et al. 2002).

LA is synthesized from oleic acid (C18:1), a long chain FA that already contains a double bond between the 9th and 10th carbon atom. Using oleic acid as a substrate, a ∆12 desaturase then introduces a second double bond between the 12th and 13th C atom (Fig. 3). In plants and yeasts genes encoding a ∆12 desaturase have been identified and characterized, for example in *Kluyveromyces lactis* (Kainou et al. 2006), *Candida parapsilosis* (Buček et al. 2014), and *Arabidopsis thaliana* (Covello and Reed 1996). In plants LA desaturation has been linked to six loci (*fad* &, *fad 2, fad 4, fad 5, fad 6* and *fad 7*) (Browse and Somerville 1991) and it was discovered that the enzyme encoding plant ∆12 desaturase contains three conservative histidine boxes belonging to the Omega family (Hashimoto et al. 2008). Until now, orthologs of these genes seem to be missing completely in most animals (except for *Caenorhabditis elegans*, see below), also in species that were found to synthesize LA (Watts and Browse 2002; Alonso et al. 2003, 2014; Blaul et al. 2014). This confirms the result from our phylogenetic analysis that LA synthesis in animals evolved secondarily, and suggests that a different genetic pathway is enabling LA synthesis in animals.

Whilst 25 arthropod species were found capable of synthesizing LA (Fig. 1), for only very few of those species a ∆12 desaturase gene or enzyme has so far been identified (Zhou et al. 2008).

*Caenorhabditis elegans* is the exception, because a plant-like ∆12 desaturase gene has been found in this species (Peyou-Ndi et al. 2000). The enzyme encoded by the gene, however, was discovered to have an alternative mechanism of desaturase functioning which relies on bifunctional activity of ∆12/∆15 desaturases (Zhou et al. 2011). Similarly, the ∆12/∆15 bifunctional desaturase was also found in the soil yeast *Lipomyces kononenkoae*, which uses a novel gene derived from gene duplication (Yan et al. 2013). However, the multiple genetic pathways that underlie LA synthesis in fungi and nematodes are unlikely to share a common evolutionary origin since a comparative phylogenetic analysis suggested that nematode Acyl-CoA desaturases probably arose independently from those found in fungi and protozoa (Zhou et al. 2011). Biochemical and genetic pathways of linoleic acid in plants and microbes are extensively reviewed in other publications (Farmer 1994; Certik and Shimizu 1999; Thelen and Ohlrogge 2002; Weber 2002; Qi et al. 2004; Sampath and Ntambi 2005; Palmquist et al. 2005; Jenkins et al. 2014).

In insects capable of LA synthesis, only two genes have so far been identified that encode a Δ12-desaturase; one in the red flour beetle, *Tribolium castaneum* and another one in the house cricket, *Acheta domestiucus* (Zhou et al. 2008). A comparative analysis showed that their Δ12-desaturase genes are more closely related to the archetypal Δ9-desaturase from rats than to Δ12-desaturases widely reported in plants (Zhou et al. 2008, 2011; Yan et al. 2013) and they most likely evolved independently from plant, fungal and nematode Δ12-desaturases. Although both species share the common ancestor of the insects, their Δ12-desaturase genes do not cluster together but they are more related to Δ9-desaturases genes of their own species, again suggesting evolution by an independent route (Zhou et al. 2008). The question arises if the close similarity to Δ9-desaturases genes is indicative of ∆9/∆12 desaturase bifunctionality similar to what has been found in *C. elegans* and some fungi. As of yet no bifunctional activity has, however, been confirmed (Zhou et al. 2008). The similarity in histidine motifs between ∆9 and ∆12 desaturases frustrates attempts to identify ∆12-desaturase genes in more species. For example, gene identification was attempted in the parasitoid *Nasonia vitripennis*, but despite several candidates no ∆12 functionality was detected in any of the genes (Blaul et al. 2014). As gene detection can be compromised by dual functionality of ∆9/∆12-desaturases (Zhou et al. 2011) and by the high similarity between ∆9 and ∆12 desaturase motifs (Zhou et al. 2008, 2011), future research should focus on those two scenarios.

### Acquisition and Utilization of LA as Drivers of LA Synthetic Ability

**Fig. 4:**
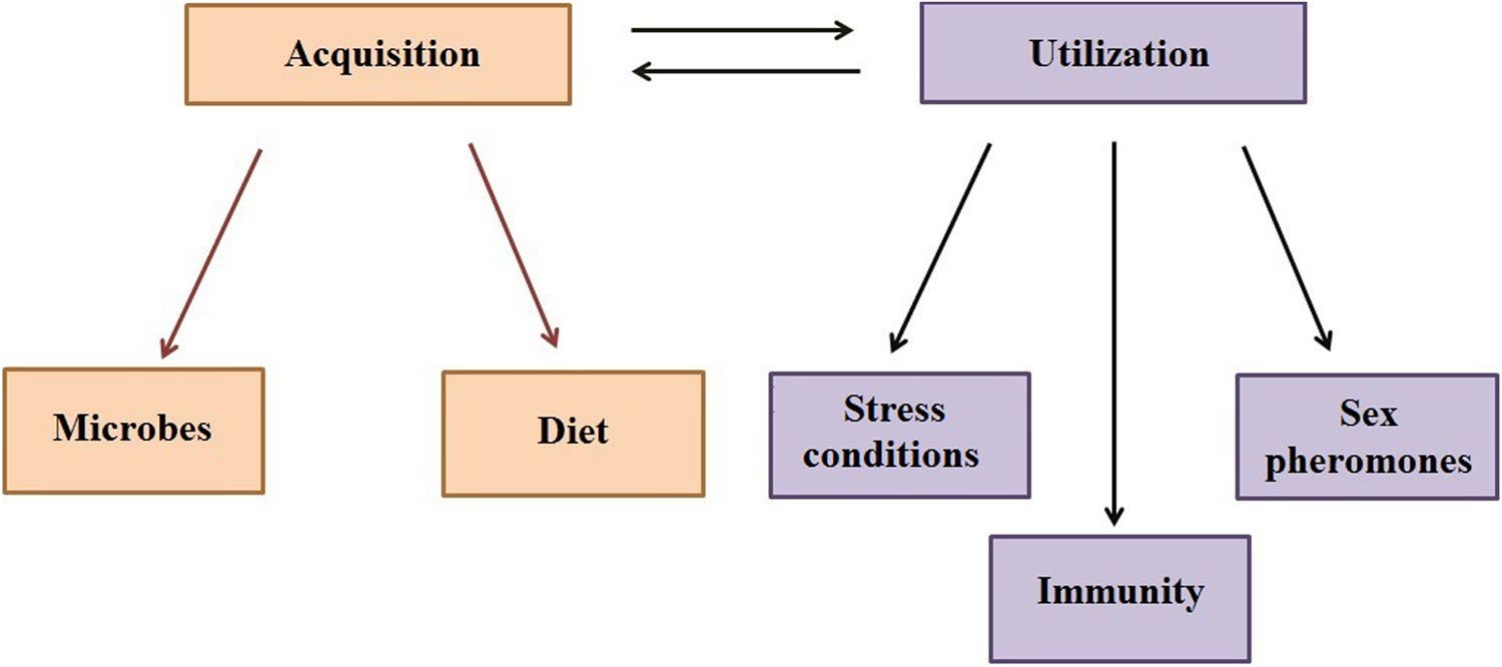
A schematic overview of the means by which LA can be acquired and utilized by organisms

Tracing the evolutionary history of a trait can reveal major evolutionary trait changes, such as gains, losses and reversions, but we need to link these transitions to the ecology of the organism to provide adaptive explanations for trait evolution. The selective forces on LA biosynthetic ability are expected to depend on the acquisition of LA from external sources and on the magnitude of LA utilization by the organism. We, therefore, need to address two key questions: (1) what are the ways in which an organism can obtain LA, and (2) to what extent does an organism utilize LA (Fig. 4)?

### Acquisition of LA from the Environment

Diet was proposed as a key ecological factor affecting LA synthesis (Cripps et al. 1986) with the evolutionary loss of LA synthesis resulting from environmental compensation (Visser et al. 2010; Ellers et al. 2012). A scenario under which the common ancestor of (primitive) insects fed on a diet rich in LA (i.e. herbivores/omnivores) could have fueled the evolutionary loss of LA synthetic ability, as the trait would have become unnecessary. Conversely, LA may be required by species that feed on a diet containing little or no LA (carnivores), leading to a regain of LA synthetic ability in these lineages (Stanley-Samuelson et al. 1991). The evolution of LA synthesis seems unrelated to dietary preferences of distinct invertebrate groups, because 50% of species capable of de novo LA synthesis are herbivores and 45% are omnivores (Table 1) (Malcicka et al. 2017). Dietary compensation could, however, still play a role in the evolution of LA synthesis. Functional requirements for LA can be highly species-specific and information on dietary fatty acid profiles could prove extremely helpful in determining the role of dietary preferences in the evolution LA synthesis. We currently have insufficient information, however, on dietary fatty acid profiles for species known to lack or synthesize LA. Future research could aim to study dietary fatty acid profiles of closely related species that differ in their ability to synthesize LA. High species specificity could further explain why some invertebrates are able to synthesize LA despite feeding on a high-LA containing diet.

Another scenario under which organisms could obtain essential nutrients is via symbioses with microbes. Ecological interactions were recently found to play a critical role in fueling the loss of essential traits, i.e. compensated trait loss (Visser et al. 2010; Ellers et al. 2012). Here, an essential function or resource is provided by the environment, usually by a symbiotic partner, rendering the trait in the receiving organism either redundant or too costly to maintain, ensuing trait loss. When the ecological partner/interaction is absent, however, the functional requirement for the trait remains; hence fitness is negatively affected in absence of the ecological partner/interaction. Symbiotic partners, such as endosymbionts, may thus play a key role in the evolution of LA synthesis.

Perhaps one of the most thorough studies into the role of microorganisms in LA synthesis was done by Borgeson et al. (1991), in which several tissues of the house cricket *Acheta domestica* and the American cockroach *Periplaneta americana* were examined under axenic (i.e. endosymbiont-free) conditions. Their results unequivocally demonstrated that the insect tissues themselves, and not microorganisms, were responsible for the synthesis of LA. Employing a slightly different methodology, the role of endosymbionts in the pea aphid, *Acyrthosiphon pisum*, has also been studied (de Renobales et al. 1986). Aphids are known to harbor different species of microbes, some of which are essential for their survival (reviewed in Douglas 1998; Oliver et al. 2010). Nevertheless, tetracycline-treated aphids with greatly reduced numbers of intracellular symbionts synthesized LA in the same proportions as untreated controls (de Renobales et al. 1986). Several other studies on insects have provided additional evidence that microorganisms are not involved in LA synthesis (Wharton and Lola 1970; Mauldin et al. 1978; Dwyer et al. 1981; Blomquist et al. 1982; Stanley-Samuelson et al. 1986a; de Renobales et al. 1986; Jurenka et al. 1987; Borgeson et al. 1991) and we have yet to discover an example of endosymbiont-mediated LA synthesis. Moreover, the discovery of Δ12-desaturase genes in *A. domesticus* and *T. castaneum* (Zhou et al. 2008) supports the above findings that microorganisms are not mediating LA synthesis.

### Utilization of LA by the Organism

Not all animal species have the same quantitative need for LA, as the biochemical and physiological pathways for which LA is used depend on the species’ biology and behavior. One of the most important functions of PUFAs is within cell membranes. Maintaining proper membrane fluidity is a crucial function of biological membranes in poikilothermic species, also known as homeoviscous adaptation (for review see Hazel 1995). This homeoviscous adaptation is achieved by desaturation of the phospholipid FA chain, which determines melting temperature and fluidity of biological membranes (Fromm and Hargrove 2012). Although homeoviscous adaptation is a very general mechanism in animals, it could be a driving force in the evolution of LA synthetic ability in organisms that regularly encounter cold temperatures or substantial temperature variation, leading to an increased demand for PUFAs. This would suggest LA synthesis to be more prevalent in temperate species encountering higher temperature variability, compared to species living in the tropics. The American and Japanese cockroaches, *P. americana* and *P. japonica*, for example, can synthesize (Z,Z)-6,9-heptacosadiene from LA, which remains as a cuticular hydrocarbon to prevent desiccation, protect against environmental chemicals, and serve as a pheromone, kairomone and defense chemical (Dwyer et al. 1981). The same compound was also found in the beetle (*Tetropium cinnamopterum*) and a hymenopteran (*Ampulex compressa*). It is unclear whether these species synthesize LA, but their phylogenetic position suggests the potential for de novo LA synthesis. The distribution of species living in temperate climates is, however, scattered on the phylogenetic tree with half of the species able to synthesize LA while the other half is not (Fig. 1). The phylogeny would thus suggest that adaptation to colder or more variable thermal environments may not be the main driving force underlying the evolution of LA synthesis. Moreover, some species, such as those regularly exposed to stressful environmental conditions, may require much higher quantities of LA compared to closely related species inhabiting more hospitable environments. For example, some species of yeast and algae were found to require more LA when exposed to lower temperature, higher salinity or nitrogen starvation (Gostinčar et al. 2009; Lu et al. 2009; Iskandarov et al. 2010; Chodok et al. 2013; Kaye et al. 2015).

Another role of LA is to modulate immune function through its effect on eicosanoid synthesis (Chuang et al. 2001; Eder et al. 2003). Eicosanoids are signaling molecules made by the enzymatic or non-enzymatic oxidation of PUFAs, which mediate specific cell actions, connect innate and adaptive immunity, and eicosanoids play a significant role in insect immune responses to bacterial, fungal and viral infections (Stanley-Samuelson et al. 1991; Harizi and Gualde 2005; Stanley et al. 2012; Büyükgüzel 2012; Park et al. 2012). Eicosanoids are also involved in egg-laying behaviors of some insects and vertebrates (Stanley 2006). For instance, the eicosanoid prostaglandin was found to mediate egg-laying behavior in some cricket species and to stimulate hatching behavior in barnacle larvae (Destephano and Brady 1977; Yamaja Setty and Ramaiah 1979; Stanley-Samuelson et al. 1986b; Stanley 2006).

A final possible way through which LA can be utilized is in the production of sex pheromones. LA has been identified as a critical component or precursor of sex pheromones in many insect species, particularly in Lepidoptera and Diptera, but also in some species of Coleoptera and Acari (Blomquist et al. 1987; Vanderwel and Oehlschlager 1987; Rule and Roelofs 1989; Blaul et al. 2014; Shimizu et al. 2014; Rong et al. 2015). In the mite *Carpoglyphus lactis* (capable of LA synthesis), for example, LA was converted to (Z,Z)-6,9-heptadecadiene, which is widely used as alarm, sex, and/or aggregation pheromone among astigmatid mites (Kuwahara 2004; Shimizu et al. 2014). A recent study further showed that the production of sex pheromones in *Nasonia vitripennis* males reared on a LA-enriched diet was four times higher than that of males reared on a minimal LA-containing diet; despite the fact this species is capable of LA synthesis (Brandstetter and Ruther 2016). Moths in the families Geometridae, Arctiidae, and Noctuidae also utilize LA as a precursor for pheromone production (Millar 2000). Although no record exists on de novo synthesis of LA in species from these families, their position within the phylogenetic tree (Fig. 1) would suggest that they cannot synthesize LA. The evolution of LA synthesis thus seems to be unrelated to pheromone production, as many species require LA as a precursor but cannot synthesize LA de novo (Table 1; Millar 2000).

### Perspective on the Evolution of LA Synthesis

The question why some organisms possess the ability for LA synthesis and other, even closely related species, do not is still open. One potential explanation might be in the quantity of LA present in the diet relative to the organism’s requirements (Nugteren et al. 1979; Domenichiello et al. 2016). LA synthesis might also depend on the interplay between a species’ ecology (how much is in the diet) and the biological functions for which LA and its derivatives are required by a certain species. Some species (e.g. yeast, algae) may require much more LA than others, depending on environmental challenges such as stress caused by fluctuations in temperature, salinity or nitrogen availability (Gostinčar et al. 2009; Lu et al. 2009; Iskandarov et al. 2010; Chodok et al. 2013; Kaye et al. 2015). Other species may require a higher amount of LA because of the functions that LA performs in egg-laying behavior, innate immune reactions to infections (viral, bacterial, and fungal), the production of sex pheromones or in the performance and survival of immature stages (Stanley 2006; Park et al. 2012; Blaul et al. 2014; Brandstetter and Ruther 2016). Species that are unable to synthesize LA should complete these functions via other ways, such as through acquisition via the diet (Stanley 2006; Eleftherianos et al. 2013).

LA synthesis does not seem to evolve in response to a single function, but rather seems to evolve through a balance between the acquisition and requirement of LA. In terms of mechanisms, LA has clear pleiotropic functions, explaining why the metabolic machinery underlying LA synthesis remains in place and why it is easy to re-evolve this phenotypic function (although some organisms seem to lack the ∆12 gene) (Alonso et al. 2003). Pleiotropy could also explain occasions where LA synthesis might seem non-adaptive (i.e. the machinery remains due to evolutionary history, but the need for LA was lost for instance due to a change in environmental conditions). Furthermore, the dual function of some ∆12 desaturases (∆12/15 in *C. elegans* and *L. kononenkoae*) suggests that the underlying gene already has a novel function (Zhou et al. 2011; Yan et al. 2013). Work on desaturase genes indeed shows that there is rapid functional divergence even within subfamilies (Hashimoto et al. 2008). This might explain why even closely related species do not share the same mechanism behind LA synthesis and why it is hard to find any general evolutionary (phylogenetic) patterns of LA synthesis mechanism between plants, microorganism and/or invertebrates.

## Conclusion

LA is involved in a wide array of biological functions and genes underlying LA synthesis show a high degree of divergence. We suggest future studies on the evolution of LA synthesis could focus on distinct taxonomic groups where closely related species vary in the ability to synthesize LA. Thorough investigation of the animal’s ecology (dietary FA profiles, sex pheromones, homeoviscous adaptation, stress and use of eicosanoids) and a search for underlying Δ12-desaturase genes could then reveal how LA acquisition and utilization interact to drive the evolution of LA synthesis.

## Electronic supplementary material

Below is the link to the electronic supplementary material.

Supplementary material 1 (DOCX 21 KB)

Supplementary material 2 (DOCX 24 KB)
