# Supplementary material for: An Evolutionary Perspective on Linoleic Acid Synthesis in Animals
Source: Evol Biol. 2017 Oct 23;45(1):15–26. doi: 10.1007/s11692-017-9436-5 (PMC5816129; doi:10.1007/s11692-017-9436-5)
Supplement: Supplementary file 1 — Supplementary material 1 (DOCX 21 KB) [file 11692_2017_9436_MOESM1_ESM.docx]

Relationships among insects and other invertebrates were inferred from the phylogeny of 1478 protein-coding genes described in Misof *et al.,* (2014). Phylogenetic relationships within orders were constructed using morphological and molecular data (Gwynne, 1995; Dohlen & Moran, 1995; Yeates & Wiegmann, 1999; Huang *et al.,* 2000; Maekawa & Matsumoto, 2000; Caterino *et al.,* 2001; Litzenberger & Chapco, 2003; Braby *et al.,* 2006; Hunt *et al.,* 2007; Cameron *et al.,* 2007; Marvaldi *et al.,* 2008; Ortiz-Rivas & Martínez-Torres, 2010; Regier *et al.,* 2012; Khaing *et al.,* 2014; Djernæs *et al.,* 2015).

**References**

Braby, M.F., Vila, R., & Pierce, N.E. (2006). Molecular phylogeny and systematics of the Pieridae (Lepidoptera: Papilionoidea): higher classification and biogeography. *Zoological Journal of the Linnean Society*, *147*(2), 239-275.

Cameron, S.L., Lambkin, C.L., Barker, S.C., & Whiting, M.F. (2007). A mitochondrial genome phylogeny of Diptera: whole genome sequence data accurately resolve relationships over broad timescales with high precision. *Systematic Entomology*, *32*(1) 40-59.

Caterino, M.S., Reed, R.D., Kuo, M.M., & Sperling, F.A. (2001). A partitioned likelihood analysis of swallowtail butterfly phylogeny (Lepidoptera: Papilionidae). *Systematic Biology*, *50*(1), 106-127.

Djernaes, M., Klass, K.D., & Eggleton, P. (2015). Identifying possible sister groups of Cryptocercidae+ Isoptera: A combined molecular and morphological phylogeny of Dictyoptera. *Molecular Phylogenetics and Evolution*, *84,* 284-303.

Gwynne, D.T. (1995). Phylogeny of the Ensifera (Orthoptera): a hypothesis supporting multiple origins of acoustical signalling, complex spermatophores and maternal care in crickets, katydids, and weta. *Journal of Orthoptera Research,* *4,* 203-218.

Huang, Y., Ortı́, G., Sutherlin, M., Duhachek, A., & Zera, A. (2000). Phylogenetic relationships of North American field crickets inferred from mitochondrial DNA data. *Molecular Phylogenetics and Evolution*, *17*(1), 48-57.

Hunt, T., Bergsten, J., Levkanicova, Z., Papadopoulou, A., John, O.S., Wild, R. et al*.* (2007). A comprehensive phylogeny of beetles reveals the evolutionary origins of a superradiation. *Science,* *318*(5858), 1913-1916.

Khaing, T.M., Shim, J.K., & Lee, K.Y. (2014). Molecular identification and phylogenetic analysis of economically important acaroid mites (Acari: Astigmata: Acaroidea) in Korea. *Entomological Research*, *44*(6), 331-337.

Litzenberger, G., & Chapco, W. (2003). The North American Melanoplinae (Orthoptera: Acrididae): a molecular phylogenetic study of their origins and taxonomic relationships. *Annals of the Entomological Society of America*, *96*(4), 491-497.

Maekawa, K., & Matsumoto, T. (2000). Molecular phylogeny of cockroaches (Blattaria) based on mitochondrial COII gene sequences. *Systematic entomology*, *25*(4), 511-519.

Marvaldi, A.E., Duckett, C.N., Kjer, K.M., & Gillespie, J.J., (2009). Structural alignment of 18S and 28S rDNA sequences provides insights into phylogeny of Phytophaga (Coleoptera: Curculionoidea and Chrysomeloidea). *Zoologica Scripta*, *38*(1), 63-77.

Misof, B., Liu, S., Meusemann, K., Peters, R.S., Donath, A., Mayer, C., Frandsen, P.B. et al*.* (2014). Phylogenomics resolves the timing and pattern of insect evolution. *Science,* 3*46*(6210), 763-767.

Ortiz-Rivas, B., & Martínez-Torres, D. (2010). Combination of molecular data support the existence of three main lineages in the phylogeny of aphids (Hemiptera: Aphididae) and the basal position of the subfamily Lachninae. *Molecular Phylogenetics and Evolution*, *55*(1), 305-317.

Regier, J.C., Mitter, C., Solis, M., Hayden, J.E., Landry, B., Nuss, M. et al. (2012). A molecular phylogeny for the pyraloid moths (Lepidoptera: Pyraloidea) and its implications for higher‐level classification *Systematic Entomology*, *37*(4), 635-656.

von Dohlen, C.D., & Moran, N.A. (1995). Molecular phylogeny of the Homoptera: a paraphyletic taxon. *Journal of Molecular Evolution*, *41*(2), 211-223.

Yeates, D.K., & Wiegmann, B.M. (1999). Congruence and controversy: toward a higher-level phylogeny of Diptera. *Annual review of entomology*, *44*(1), 397-428.
