# Supplementary material for: An Evolutionary Perspective on Linoleic Acid Synthesis in Animals
Source: Evol Biol. 2017 Oct 23;45(1):15–26. doi: 10.1007/s11692-017-9436-5 (PMC5816129; doi:10.1007/s11692-017-9436-5)
Supplement: Supplementary file 2 — Supplementary material 2 (DOCX 24 KB) [file 11692_2017_9436_MOESM2_ESM.docx]

1. Rothstein, M., & Götz, P. (1968). Biosynthesis of fatty acids in the free-living nematode, *Turbatrix aceti*. *Archives of Biochemistry and Biophysics*, *126*(1),  131-140.

2. Chitwood, D.J., & Krusberg, L.R. (1981). Diacyl, alkylacyl, and alkenylacyl phospholipids of *Meloidogyne javanica* females*. Journal of Nematology*, *13*(2), 105.

3. Fodor, A., Dey, I., Farkas, T., & Chitwood, D.J. (1994). Effects of temperature and dietary lipids on phospholipid fatty acids and membrane fluidity in *Steinernema carpocapsae*. *Journal of Nematology*, *26*(3), 278.

4. Wallis, J.G., Watts, J.L., & Browse, J. (2002). Polyunsaturated fatty acid synthesis: what will they think of next? *Trends in Biochemical Sciences*, *27*(9), 467-473.

5. Aboshi, T., Shimizu, N., Nakajima, Y., Honda, Y., Kuwahara, Y., Amano, H. et al. (2013). Biosynthesis of linoleic acid in *Tyrophagus* mites (Acarina: Acaridae). *Insect Biochemistry and Molecular Biology*, *43*(11), 991-996.

6. Shimizu, N., Naito, M., Mori, N., & Kuwahara, Y. (2014). *De novo* biosynthesis of linoleic acid and its conversion to the hydrocarbon (Z, Z)-6, 9-heptadecadiene in the astigmatid mite, *Carpoglyphus lactis*: Incorporation experiments with sup 13 sup C-labeled glucose. *Insect Biochemistry and Molecular biology*, *45*, 51-57.

7. Weinert, J., Blomquist, G.J., & Borgeson, C.E. (1993). *De novo* biosynthesis of linoleic acid in two non-insect invertebrates: the land slug and the garden snail. *Cellular and Molecular Life Sciences*, *49*(10), 919-921.

8. Cripps, C., Blomquist, G.J., & De Renobales, M. (1986). *De novo* biosynthesis of linoleic acid in insects. *Biochimica et Biophysica Acta (BBA)-Lipids and Lipid Metabolism*, *876*(3), 572-580.

9. Mauldin, J.K., Smythe, R.V., & Baxter, C.C. (1972). Cellulose catabolism and lipid synthesis by the subterranean termite, *Coptotermes formosanus*. *Insect Biochemistry*, *2*(6),209-217.

10. Blomquist, G.J., Dwyer, L.A., Chu, A.J., Ryan, R.O., & De Renobales, M. (1982). Biosynthesis of linoleic acid in a termite, cockroach and cricket. *Insect Biochemistry*, *12*(3), 349-353.

11. Borgeson, C.E., Kurtti, T.J., Munderloh, U.G., & Blomquist, G.J., (1991). Insect tissues, not microorganisms, produce linoleic acid in the house cricket and the American cockroach. *Cellular and Molecular Life Sciences*, *47*(3), 238-241.

12. Worthington, R.E., Brady, U.E., Thean, J.E., & Wilson, D.M., (1981). Arachidonic acid: occurrence in the reproductive tract of the male house cricket (*Acheta domesticus*) and field cricket (*Gryllus spp*.).  *Lipids*, *16*(1),79-81.

13. Stanley-Samuelson, D.W., & Loher, W. (1986). Prostaglandins in insect reproduction. *Annals of the Entomological Society of America*, *79*(6), 841-853.

14. Jurenka, R.A., Stanley-Samuelson, D.W., Loher, W., & Blomquist, G.J. (1988). *De novo* biosynthesis of arachidonic acid and 5, 11, 14-eicosatrienoic acid in the cricket *Teleogryllus commodus. Biochimica et Biophysica Acta (BBA)-Lipids and Lipid Metabolism*, *963*(1), 21-27.

15. Bade, M.L. (1964). Biosynthesis of fatty acids in the roach *Eurycotis floridana*. *Journal of Insect Physiology*, *10*(2), 333-341.

16. Louloudes, S.J., Kaplanis, J.N., Robbins, W.E., & Monroe, R.E., (1961). Lipogenesis from C14-acetate by the American cockroach. *Annals of the Entomological Society of America*, *54*(1),  99-103.

17. Dwyer, L.A., Blomquist, G.J., Nelson, J.H., & George Pomonis, J. (1981). A sup 13 sup C-NMR study of the biosynthesis of 3-methylpentacosane in the American cockroach. *Biochimica et Biophysica Acta (BBA)-Lipids and Lipid Metabolism*, *663*(2), 536-544.

18. Jurenka, R.A., De Renobales, M., & Blomquist, G.J. (1987). *De novo* biosynthesis of polyunsaturated fatty acids in the cockroach *Periplaneta americana. Archives of Biochemistry and Biophysics*, *255*(1),184-193.

19. De Renobales, M., Ryan, R.O., Heisler, C.R., McLean, D.L., & Blomquist, G.J. (1986). Linoleic acid biosynthesis in the pea aphid, *Acyrthosiphon pisum* (Harris). *Archives of Insect Biochemistry and Physiology*, *3*(2),193-203.

20. Strong, F.E. (1963). Fatty acids: in vivo synthesis by the green peach aphid, *Myzus persicae* (Sulzer). *Science* , *140*(3570), 983-984.

21. Greenway, A.R., Griffiths, D.C., Furk, C., & Prior, R.N.B. (1974). Composition of triglycerides from aphids of six different families and from different seasonal forms of *Aphis evonymi. Journal of Insect Physiology*, *20*(12), 2423-2431.

22. Buckner, J.S., & Hagen, M.M. (2003). Triacylglycerol and phospholipid fatty acids of the silverleaf whitefly: composition and biosynthesis. *Archives of Insect Biochemistry and Physiology*, *53*(2), 66-79.

23. Blaul, B., Steinbauer, R., Merkl, P., Merkl, R., Tschochner, H., & Ruther, J. (2014). Oleic acid is a precursor of linoleic acid and the male sex pheromone in *Nasonia vitripennis*. *Insect Biochemistry and Molecular Biology*, *51*, 33-40.

24. Zhou, X.R., Horne, I., Damcevski, K., Haritos, V., Green, A., & Singh, S. (2008). Isolation and functional characterization of two independently‐evolved fatty acid Δ12‐desaturase genes from insects.  *Insect Molecular Biology*, *17*(6), 667-676.

25. Nwanze, K.F., Maskarinec, J.K., & Hopkins, T.L. (1976). Lipid composition of the normal and flight forms of adult cowpea weevils, *Callosobruchus maculatus. Journal of Insect Physiology*, *22*(6), 897-899.

26. Baker, J.E., & Nelson, D.R. (1981). Cuticular hydrocarbons of adults of the cowpea weevil, *Callosobruchus maculatus.*  *Journal of Chemical Ecology*, *7*(1), 175-182.

27. Turunen, S. (1975). Absorption and transport of dietary lipid in *Pieris brassicae. Journal of Insect Physiology*, *21*(8), 1521-1529.

28. Cenedella, R.J., (1971). The lipids of the female monarch butterfly, *Danaus plexippus*, during fall migration. *Insect Biochemistry*, *1*(2), 244-247.

29. Lambremont, E.N., & Dial, P.F. (1980). Fatty acid composition of major phospholipids from the fat body, flight muscle and testis of the house cricket, and positional distribution of fatty acids in testicular phospholipids. *Comparative Biochemistry and Physiology Part B: Comparative Biochemistry*, *66*(2), 327-330.

30. Nelson, D.R., & Sukkestad, D.R. (1968). Fatty acid composition of the diet and larvae and biosynthesis of fatty acids from 14 C-acetate in the cabbage looper, *Trichoplusia ni. Journal of insect Physiology*, *14*(2), 293-300.

31. Keith, A.D. (1967). Fatty acid metabolism in *Drosophila melanogaster*: Interaction between dietary fatty acids and *De novo* synthesis. *Comparative biochemistry and physiology*, *21*(3), 587-600.

32. Madariaga, M.A., Mata, F., Municio, A.M., & Ribera, A. (1972). Effect of the lipid composition of the larval diet on the fatty acid composition during development of *Ceratitis capitata.*  *Insect Biochemistry*, *2*(7), 249-256.

33. Madariaga, M.A., Mata, F., Municio, A.M., & Ribera, A. (1974). Changes in the fatty acid patterns of glycerolipids of *Dacus oleae* during metamorphosis and development. *Insect Biochemistry*, *4*(2), 151-160.

34. Castillon, M.P., Jimenez, C., Catalan, R.E., & Municio, A.M. (1971). Biochemistry of the development of the fly *Ceratitis capitata*: Evolution of fatty acids of individual phospholipids. *Insect Biochemistry*, *1*(3), 309-315.

35. Municio, A.M., Lizarbe, M.A., Relaño, E., & Ramos, J.A. (1977). Fatty acid synthetase complex from the insect *Ceratitis capitata. Biochimica et Biophysica Acta (BBA)-Lipids and Lipid Metabolism*, *487*(1),175-188.
